# Combination of laser-generated silicon NPs and electrospun-nanofibres for the development of new-generation bioactive wound dressings

**DOI:** 10.1039/d5ra09481j

**Published:** 2026-01-02

**Authors:** Romain Scarabelli, Magali Gary-Bobo, Christophe Nguyen, Denis Durand, Jerôme Esvan, Maëlenn Aufray, Christophe Drouet, Ahmed Al-Kattan

**Affiliations:** a LP3, Aix Marseille Université, CNRS, UMR 7341 Campus de Luminy, Case 917 13288 Marseille France ahmed.al-kattan@univ-amu.fr; b CIRIMAT, Université de Toulouse/INP/CNRS 4 allée Emile Monso 31030 Toulouse France; c IBMM, Université de Montpellier, CNRS, ENSCM 1919 Route de Mende 34293 Montpellier France

## Abstract

The fabrication of wound dressings able to promote and accelerate healing is a key challenge to manage a variety of complex wounds including chronic disease or traumatic wounds. In this context, electrospun-nanofibrous scaffolds involving bioactive nanoparticles (NPs) appear as a very promising approach, which was investigated in the present work. We report the fabrication and evaluation of novel bioactive wound dressings associating electrospun ε-polycaprolactone (PCL) imbricated nanofibers (NFs) and laser-synthesized silicon NPs (SiNPs) obtained from green, impurity-free physical routes. A successful protocol of PCL NFs preparation and functionalization with SiNPs was achieved in aqueous acidic solution in the presence of aminopropyltriethoxysilane (APTES). We demonstrated this proof of concept by (1) assessing the effect of the main compositional and process parameters (high voltage, flow rate, collector-injector distance) to yield reproducible NFs, (2) verifying by FTIR the chemical stability of the PCL in the processing conditions, and (3) unveiling the key role of APTES used in the NFs functionalization and their successful association with NPs. On the basis of HR-SEM observations, NFs showed uniform SiNPs distribution throughout the fibers with structural stability in physiological medium. XPS and TEM analyses allowed investigating the SiNPs, and their chemical composition was mainly metallic Si with only top-surface oxidation. Then, physico-chemical, mechanical and bioactivity properties of the NFs were evaluated at three increasing concentrations of SiNPs. All hybrid NFs-APTES-NPs formulations prepared were evaluated from an *in vitro* biological point of view using two cell types relevant to our final wound healing applications, namely human HaCaT keratinocytes and murine C2C12 myoblasts. In all cases, no cytotoxicity was detected for any of the developed biomaterials, and a proliferative role of the SiNPs was unveiled. The absence of detectable inflammatory potential for all biomaterials tested was also assessed *via* Griess assays, with even a mitigation effect of the inflammatory response by the NPs. Based on these biological results, an optimized bioactive wound dressing formulation was developed, showing high potential for the management of complex wounds combining biocompatibility, pro-healing capabilities and eco-friendly production. The work opens up exciting perspectives towards the design of efficient bioactive wound dressings based on hybrid electrospun-nanofibers functionalized with laser-synthesized SiNPs.

## Abstract 2

The fabrication of wound dressings able to promote and accelerate healing is a key challenge to manage a variety of complex wounds including chronic disease or traumatic wounds.

## Graphical abstract

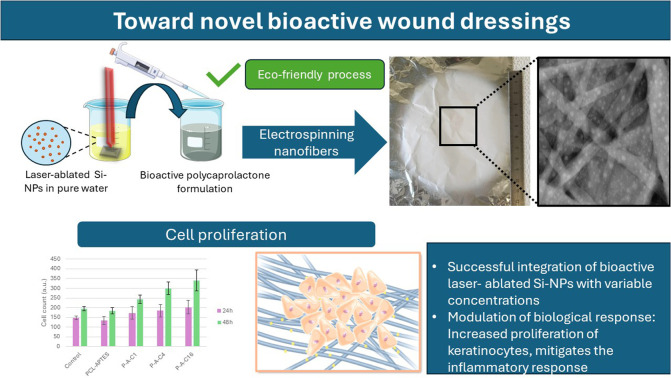

## Introduction

The fabrication of wound dressings able to promote and accelerate healing is still a key challenge to manage a variety of complex wounds including in chronic diseases, traumatic wounds, and immune-depressed patients. Traditional wound dressings including cotton bandages and gauze remain widely used in medical practice. However, although effective as physical barriers, they lack bioactivity to favor tissue healing and need to be replaced frequently, which may cause additional local lesions or even facilitate infections.^1,2^ Alternative wound dressings based on hydrocolloids, hydrogels or alginate compositions have been developed to maintain a moist environment and to absorb exudates from the open wound. However, excessive fluid retention may favor bacterial colonization.^2^ Thus, they need to be used by trained medical practitioners with extensive care and wound cleaning procedures, which cannot always be achieved for example in the context of emergency traumatology. In this view, the design of pro-healing wound dressing devices remains an unmet medical need. Due to their rather biomimetic structure resembling natural extracellular matrices (ECM), electrospun polymer nanofibers (NFs) have emerged as promising materials in the field of wound repair.^3^ Indeed, these fibrous structures can provide relevant support to enhance cell adhesion and proliferation. Moreover, their high surface area combined with inter-fiber porosity can facilitate the efficient diffusion of nutrients.^4^ Furthermore, the versatility of NFs can offer great interest to bring synergetic effects in view of ultimately combining hemostatic^5^ and antibacterial properties,^6^ especially thanks to the possibility to combine these fibrous (bio)polymers with biomolecules/drugs or nanoparticles (NPs).^7,8^

Among the different synthetic polymers explored for electrospinning, ε-polycaprolactone (PCL) is a widely used biocompatible and FDA-approved polymer that has received extensive interest for medical, implantable, and wound healing applications.^9,10^ This polymer also presents a slow degradation rate and high mechanical stability even in moist environments, or directly implanted *in vivo*.^11,12^ Moreover, PCL presents excellent processability through the electrospinning technique, and its surface chemistry allows for fiber functionalization using various bioactive agents, such as NPs.^13^ These characteristics position PCL as an ideal candidate for the development of new-generation wound dressings incorporating bioactive NPs.

However, to this day, most of the NPs explored in combination with PCL NFs were obtained from multistep chemical processes implying the use of various reagents, stabilizers and solvents, potentially harmful to the human body and limiting their industrial scale biomedical applications.^14^

Driven by its flexibility and speed, we have previously introduced an ultra-short femtosecond (fs) laser ablation approach in liquid state, as a relevant process to design ultra-pure crystalline NPs for biomedical applications.^15–18^ Thanks to the interaction of a fs laser beam with a solid target initially immersed in a liquid, this method allows for the formation of ultra-pure and very stable colloidal NPs. Moreover, by playing with several parameters of the laser (*e.g.*, fluence, beam time duration, ablation/fragmentation, *etc.*), we have demonstrated in preliminary studies the possibility to monitor their physicochemical properties including their size distribution and chemical composition.

Among several laser-synthesized NPs, SiNPs (silicon) appear as promising candidates to be explored as bioactive agents embedded into PCL NFs. Indeed, beside their ability to be used as efficient theranostic tools as we have shown before,^19^ SiNPs are biocompatible and biodegradable, made from silicon element which is naturally present in the human body.^20,21^ Moreover Si plays an active role in collagen production, a key component for tissue remodeling;^22–24^ and promotes platelet-endothelial tissue interaction, which is also relevant for hemostasis and wound healing applications.^25^ Very recently, we have also proved the ability of SiNPs to promote the proliferation and differentiation of myoblasts.^15^ By incorporating SiNPs into PCL NFs, we thus hypothesize that functionalizing of NFs may enhance cell proliferation and tissue regeneration, leading to a pro-healing effect of the developed dressing.

In this context, in the present work, we aimed to develop a novel bioactive pro-healing wound dressing designed by originally combining PCL NFs with laser synthesized SiNPs. The preparation and functionalization of the PCL NFs with SiNPs will be investigated here in aqueous acidic solution and in presence of aminopropyltriethoxysilane (APTES), to develop an environmentally friendly protocol. APTES was here selected as amino-silane linker between the PCL NFs and the SiNPs basing our results on core–shell NP functionalization using APTMS.^17^ A similar approach can be encountered in the literature for the grafting of NPs or metals onto nanofibers, but generally for waste cleaning purposes.^26–28^ APTES has also been employed in wound-healing applications with no adverse effects, although usually through dipping techniques or pre-grafting of NPs, adding complexity to the product synthesis. In our case, we propose here a “one-pot” protocol, to simplify the process and allow for high tunability enabling eventual large-scale production.^29–32^

Various parameters in terms of polymer solution (*e.g.* dissolved polymer mass) and electrospinning conditions (high voltage, flow rate, collector-injector distance) will be checked, to obtain uniform PCL-NPs hybrids. In this contribution, after assessing the main physico-chemical characteristics of the PCL-NFs hybrids prepared, various *in vitro* biological properties will be investigated on the optimized dressing compositions, in terms of cytocompatibility/proliferation of murine myoblasts and human keratinocytes, and the related (non)inflammatory response.

## Materials and methods

### Materials

ε-Polycaprolactone (PCL) with an average molecular weight of 80 000 Da and aminopropyltriethoxysilane (APTES) were ordered from Sigma Aldrich (Germany). Glacial acetic acid and pure ethanol were bought from Fisher Chemicals (France). Ultrapure water was obtained using a MilliQ water purifier. Silicon wafers were ordered from Goodfellow (UK).

### Laser-ablative synthesis of Si NPs and characterization

Si-NPs were synthesized using a femtosecond laser (Light Conversion Carbide CB1-05 Yb:KGW) and a Si wafer target, at a pulse duration of 400 fs, delivering 75.8 µJ per pulse, and with a repetition rate of 10 kHz at 1028 nm. The Si wafer was placed at the bottom of a beaker, submerged by 10 mL of ultrapure water, and submitted to the laser beam for 10 min. A scanner system coupled with the laser beam permitted the focalization of the laser on the surface of the wafer, granting optimal ablation. The ablation pattern was designed as a square surface of 5 × 5 mm, with a scanning speed of 2.00 m s^−1^ to avoid overlapping in the ablation points. The SiNPs crystallographic properties were characterized with a high-resolution transmission electron microscope (HR-TEM, JEOL JEM 3010) working in imaging and diffraction modes.

To prepare the different NP concentrations, the initial SiNP suspension (C1) was centrifuged at 14 000 rpm for 15 minutes and the adequate amount of supernatant was discarded. The pellet was then redispersed in the remaining liquid using an ultrasound bath for 15 minutes. By discarding three quarters of the supernatant from C1, we obtained a fourfold concentrated suspension named C4. The same process was repeated upon C4 to yield C16. The concentration of the SiNPs was measured by inductively coupled plasma mass spectrometry (ICP-MS), resulting in 14 µg mL^−1^ for C1, 56 µg mL^−1^ for C4 and 224 µg mL^−1^ for C16. SiNPs hydrodynamic diameter in solution and zeta potential were assessed using a Zetasizer Nano ZS instrument (Malvern Instruments).

### XPS analysis

XPS measurements were carried out using a Thermo Scientific K-alpha instrument. Photoelectron emission spectra were recorded using Al-Kα radiation (*hν* = 1486.6 eV) from a monochromatized source, with an X-ray spot size of 400 µm. The pass energy was set to 30 eV (0.1 eV step) for high-resolution scans and 100 eV (1 eV step) for survey scans. A flood gun was employed to neutralize charging effects. Measurements were done on freeze-dried SiNPs suspension after several cycles of vacuum pumping to remove trace moisture.

### Preparation of the electrospinning solutions and functionalization with laser synthesized SiNPs

In comparison to our previous work, PCL beads were solubilized in an aqueous acetic acid solution, following an eco-friendly protocol. Briefly, each electrospinning solution of PCL was elaborated by mixing PCL beads in a solution of glacial acetic acid and ultra-pure water (or an aqueous suspensions of Si-NPs) in a 9 : 1 ratio. The final fraction of PCL in the liquid was 17% w/v for a total volume of 6 mL. The mixture was brought to 65 °C under vigorous stirring for 1 h until complete polymer dissolution. The functionalization of PCL with SiNPs has been achieved by using APTES at a volume fraction of 1.7%, which has been optimized to ensure functionalization and electrospinnability.

### Electrospinning process and fiber characterization

The polymer solution was poured into a 5 mL Luer-lock plastic syringe which was then placed in a pump and connected to the electrospinning device (Spinbox, Bioinicia Fluidnatek, Spain). Electrospinning parameters such as collector distance and applied voltage were explored to generate uniform NFs. Throughout all experiments, the feed rate was fixed at 0.005 mL min^−1^. All experiments were conducted at room temperature. The obtained nanofibers were observed through SEM (Jeol JSM-6390) and HR-SEM (Jeol JSM-7900F) working at 10 and 30 kV respectively. NF width measurements were made using ImageJ software, measuring over 150 individual NF over multiple micrographs for each sample. For complementary analyses on the chemical nature of the fibres, FTIR spectra were recorded using a Bruker Vertex 70 spectrometer in the 80–4000 cm^−1^ range, using 100 scans and a resolution of 4 cm^−1^.

### Digital evaluation of the porosity of the NF membranes

The porosity of the NFs samples was evaluated digitally from SEM micrographs using ImageJ, following a described procedure.^33^ To summarize, the pictures were converted in grayscale and their contrast and brightness was automatically adjusted. The images were processed using the software's native algorithm. A Gaussian blur was then applied to homogenize the surface of the NFs. A threshold of 70% was applied, selecting the upper and intermediate layers of the NFs structure, and transforming the image in black and white. The porosity of the sample was calculated using the following equation:
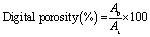
where *A*_p_ is the area of the pores and *A*_t_ the total area of the pictures. The DP was evaluated from multiple pictures of each sample.

### Water uptake test

Square samples of 2 × 2 cm of electrospun NFs were cut, lifted from their aluminum foil support, and placed in Eppendorf tubes filled with 50 mL of deionized water. After each timepoint, the samples were removed from the tubes and surface water was eliminated by lightly tapping absorbent lint-free paper on the samples. The electrospun mats mass was measured, and the water uptake was calculated using the following equation:
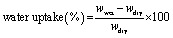
where *w*_wet_ is the weight of the wet sample and *w*_dry_ is the weight of the dry sample, before the experiment. The samples were then air-dried at room temperature overnight and weighed again to control for dry mass variation.

### Stability test in phosphate buffered saline (PBS)

To evaluate the nanofiber dressings stability in aqueous media, 2 × 2 cm samples were cut and placed in Eppendorf tubes filled with 15 mL of PBS. After 1, 3 and 7 days, each sample was removed from the PBS, rinsed with deionized water, and dried overnight at room temperature. The samples were then weighed to control for mass variation and observed using SEM.

### Mechanical properties evaluation of the nanofibrous samples

Rectangular samples of 3 × 1 cm of electrospun NFs were cut with a scalpel, following a plastic template. The membranes were then placed lengthwise in the vice grips of an INSTRON tensile machine (model 3367, INSTRON SA, France) equipped with a 500 N load sensor. Test speed was set to 10 mm min^−1^ and the stress–strain curves were recorded, presenting two quasi-linear regions; one between 0 to 5% strain, and the second between 20 to 60% strain. Calculating the slope of the linear regressions of these two regions allowed us to obtain the apparent Young's modulus and apparent yield modulus, respectively.

### Nanofibrous sample preparation for biological testing

Square samples of 2 × 2 cm of electrospun fibers were deposited in 48-well plates using 3D-printed PLA tubes for support, and soaked in ethanol for sterilization at least for 30 min. The plates were left to dry under sterile atmosphere, and then washed with PBS.

### *In vitro* cell cultures

Murine myoblasts C2C12 (ATCC) were cultured according to a previously published protocol.^15^ The medium consisted of DMEM GlutaMAX (Gibco) supplemented with 10% heat inactivated Foetal Bovine Serum (Euroclone), penicillin (100 IU mL^−1^, Gibco) and streptomycin (100 mg mL^−1^, Gibco). Healthy human keratinocytes HaCat (ATCC) were cultured in PromoCell's Keratinocyte Base Medium 2, supplemented with penicillin (100 IU mL^−1^, Gibco) and streptomycin (100 mg mL^−1^, Gibco). The cells and media were placed in T-75 flasks (Sarstedt) at 37 °C and 5% CO_2_. The images and counting of the cells were obtained using a reverse microscope (EVOS XL Core, Invitrogen, Thermo Fisher, USA).

### Cell survival assay using the ISO 10993-5 method

Following the described and standardized ISO 10993-5 method for evaluating cytocompatibility of medical devices,^34^ 700 µL of culture medium was placed in each well of a 48-well plate containing the samples, which was then put in the incubator for 48 h. Separately, murine myoblast C2C12 were seeded into 48-well plates at 10 000 cells per well, and 700 µL of culture medium (DMEM, 10% FBS, 1% PS) per well. After 48 h, the culture medium from the cells-containing wells was then replaced by the culture medium extracted from the wells containing the nanofibrous samples. The control wells received fresh, untampered culture medium. Cell viability was then determined by standard MTT colorimetric assay. The formazan crystals were dissolved in a 1 : 1 mixture of Ethanol/DMSO. The absorbance was measured at 570 nm.

### Cell counting by observation

Nanofibrous samples were placed in 48-well plates as described previously. HaCat human keratinocyte cells were seeded at a density of 50 000 cells per well and Keratinocyte Base Medium 2 was used as medium. The plate was placed in the incubator at 37 °C and 5% CO_2_, and pictures were taken after 24 and 48 h using an EVOS XL Core reverse microscope for hand-counting. Control wells contained only cells and culture medium.

### Griess inflammation assay

The membrane samples were prepared as previously described and seeded with 50 000 HaCat cells in a 48-well plate. Control wells contained only cells and culture medium. After 48 h of incubation, 250 µL of the medium was sampled and mixed with 250 µL of the Griess reagent (1% sulfanilamide and 0.1% naphthylethylenediamine dihydrochloride in 2.5% phosphoric acid) before being covered from light for 15 min. Each 500 µL sample/reagent mix was then placed in a UV/vis microcuvette and absorbance was measured at 540 nm. For statistical analysis, each absorbance point was then converted to nitrite concentration using a previously established calibration curve ranging from 0 to 100 µM.

### Statistical analysis

All experiments were performed at least in triplicate. Data were statistically analysed using one-factor ANOVA and the adequate multiple comparison tests. A *P*-value <0.05 was considered as statistically significant.

## Results and discussion

### Production and characterization of PCL nanofibers

In a first step, we focused on the production by electrospinning of NFs made of pure PCL, to serve as reference for the study. Using our previous results as a starting point,^35,36^ we have established a novel eco-friendly protocol to prepare PCL solution by dissolving the PCL beads in an acetic acid solution (see details in the Experimental section). The acetic acid/deionized water ratio, percentage of PCL in the solution, temperature and dissolution time were optimized to ensure full homogeneous PCL bead dissolution. The electrospinning parameters were also optimized by varying the applied tension and collector distance, in order to minimize the number of defects such as polymer bead or spindle-like formation beside the fibres, and to homogenize the average diameter of the fibres themselves (Table 1).

**Table 1:** Electrospinning parameters optimization for pure PCL nanofibers, and average diameter size obtained. Fixed parameters: 10 min duration and 0.005 mL min^−1^ flow rate

Collector distance (cm)	Applied voltage (kV)	Presence of polymer beads	Average fibers diameter (nm)
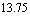	10	+	306 ± 45
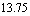	13.5	+	279 ± 65
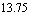	15.5	−	277 ± 58
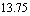	20	+	291 ± 78
14.25	15.5	−	264 ± 50
14.25	20	+	298 ± 75
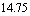	15.5	+	232 ± 62

All NFs samples were characterized by SEM observations. During the optimization process, we started our investigations by using our previously explored parameters, namely 12.75 cm and 11.5 kV. This set allowed us to obtain extremely thin NFs (averaging 207 nm) but presenting a large number of undesirable beads, likely due to imbalance between the axisymmetric and whipping instabilities of the jet. In fact, the wave-like deformation induced by the axisymmetric mode has been described as a source of defects in NFs.^37–40^ In order to favor the whipping motion of the polymer jet, and thus reduce the number of beads,^37^ the distance was then increased to 13.75 cm, and applied voltages were tested ranging from 10 to 20 kV by increments of 0.5 kV. A voltage of 15.5 kV was found to produce beadless and homogeneous NFs with an average diameter of 277 ± 58 nm. To further decrease the NFs size, the distance was increased by 0.5 cm and voltages were tested again.

**Fig. 1:**
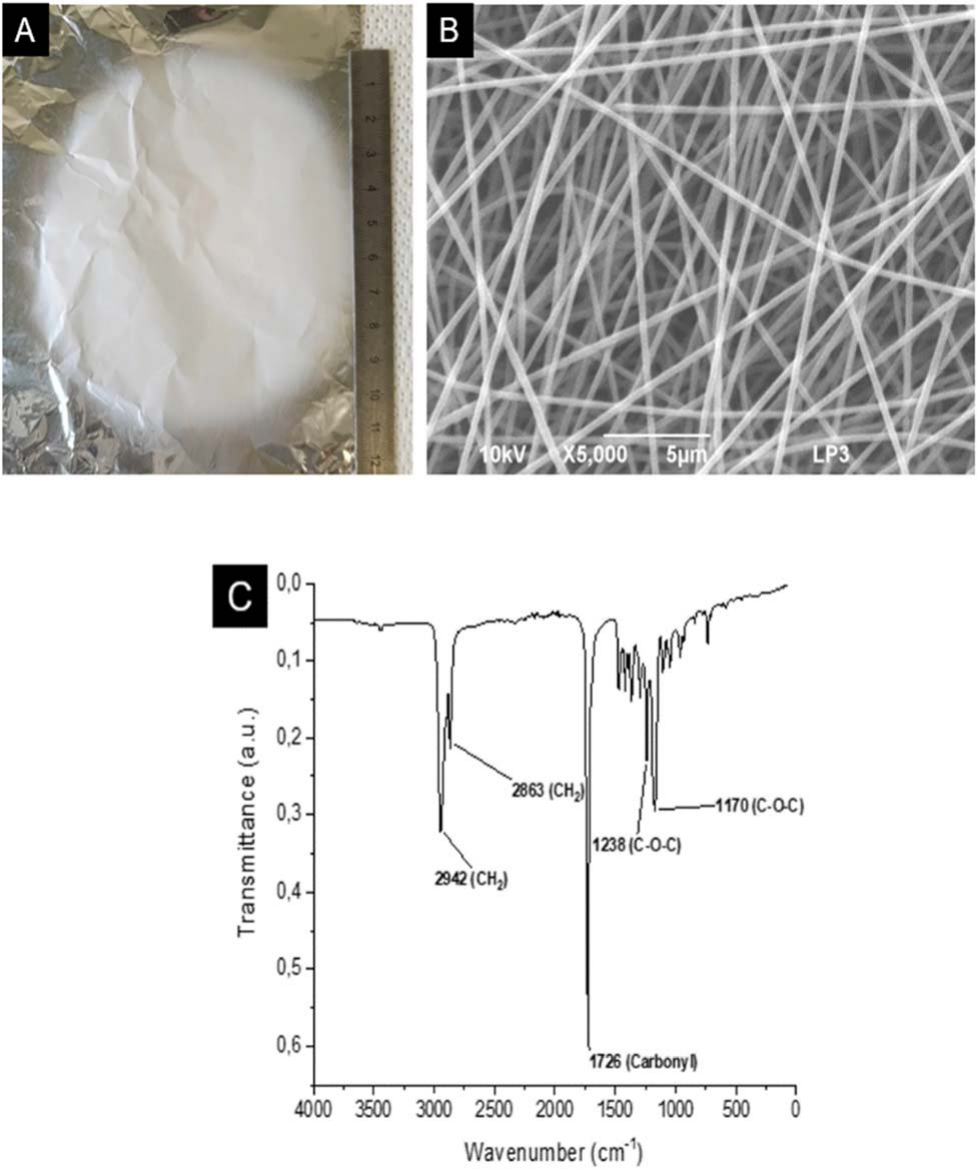
(A) Macroscopic view of the (NPs-free) electrospun nanofibrous membrane, (B) SEM microscopy observation, (C) FTIR spectrum of pure PCL electrospun nanofibers with the main bands assignments.

The optimal parameters were determined to be 14.25 cm and 15.5 kV, yielding a characteristic mat of NFs (Fig. 1A) presenting an average size of 264 ± 50 nm, assessed from SEM micrograph analysis using ImageJ as seen on Fig. 1B.

The related IR spectrum (Fig. 1C) was also acquired to check that the PCL vibrational signature was retained after the electrospinning process. Results obtained were consistent with the literature,^41,42^ as characteristic bands for PCL were found as follows: asymmetric CH_2_ stretching (2942 cm^−1^), symmetric CH_2_ stretching (2863 cm^−1^), carbonyl stretching (1726 cm^−1^), asymmetric C–O–C stretching (1238 cm^−1^) and symmetric C–O–C stretching (1170 cm^−1^).

The NFs developed here (in the absence of SiNPs) will serve as control sample for the rest of the study, with the aim to unveil the added “bioactive” value (*e.g.* the modulation of biological response towards pro-healing properties) of the NPs inclusion in the device. Prior to attempting to prepare the PCL-NPs hybrids, a special attention was first given to the generation of the SiNPs themselves, through pulsed laser ablation in liquid media (PLAL).

### Production and characterization of laser-synthesized silicon NPs

As we mentioned in the introduction, laser-synthesised NPs exhibit several benefits over chemical methods to elaborate ultra-pure Si functional NPs free from residual impurity and organic stabilizing molecules. Moreover, we have also shown that such SiNPs can play a crucial role in favoring cell proliferation. Following the protocol described in the Experimental section, the colloidal suspensions of SiNPs were produced by means of a femtosecond laser. Laser ablation in water allowed us to produce homogeneous, size-controlled metallic NPs where only a small portion of the outer layer of the NPs is oxidized, as was described in a previous study.^43^

The colloidal suspensions synthesized were firstly characterized using HR-TEM and X-ray diffraction. Exploration work was conducted to determine the ideal parameters to produce uniform, homogenous and spherical SiNPs. Considering the future use of these NPs, *i.e.* their inclusion in NFs, we aimed for an average diameter between 15 and 100 nm, to ensure that the particles' size did not exceed the diameter of the fibres, while not producing too small NPs to limit eventual sources of toxicity.^44–46^

**Fig. 2:**
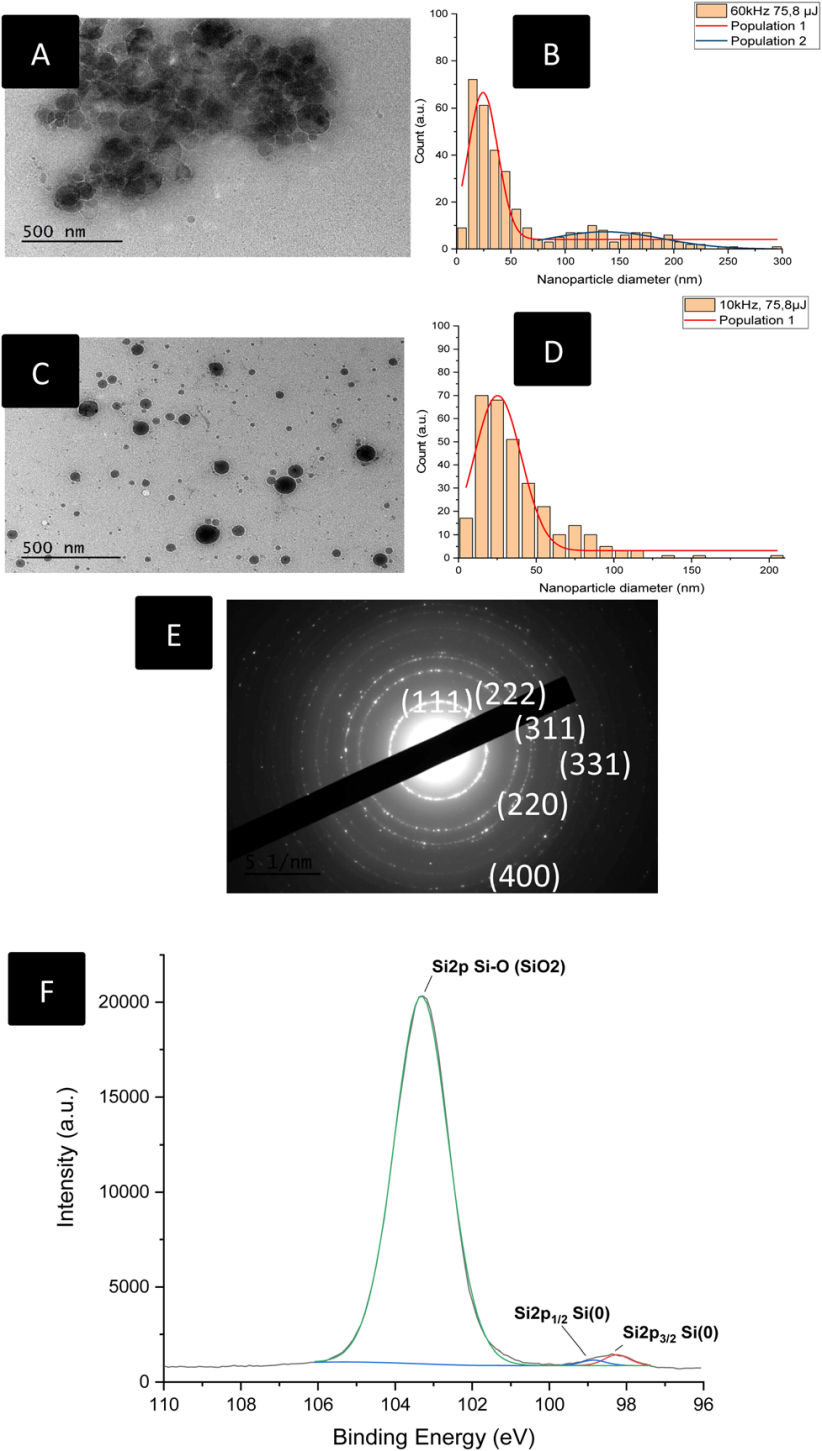
Size distributions and TEM analysis of Si-NPs produced *via* laser ablation in water. (A) TEM photograph of Si-NPs obtained with 60 kHz, 75.8 µJ; (B) size distribution of Si-NPs obtained with 60 kHz, 75.8 µJ; (C) TEM photograph of Si-NPs obtained with 10 kHz, 75.8 µJ; (D) size distribution of Si-NPs obtained with 10 kHz, 75.8 µJ; (E) electron diffraction rings of Si-NPs for ablation parameters: 10 kHz, 400 fs, 75.8 µJ per pulse. (F) High resolution XPS spectrum of the Si2p signals of the SiNPs.

As shown in Fig. 2, while optimizing the parameters, we observed more aggregation in the samples obtained at 60 kHz, as well as less homogenous samples and very distinct multiple NP size populations. At 75.8 µJ per pulse, a frequency of 60 kHz led to two distinct populations of NPs, the smaller one with an average diameter of 25 nm, and the larger one with an average of 136 nm (Fig. 2A and B). Increasing the pulse energy to up to 80 µJ produced two different populations of smaller sizes overall, 17 nm and 87 nm respectively, but a higher population of wider NPs (Fig. S3). The most homogenous and dispersed population of Si-NPs with an average diameter of 30 nm, well within the desired diameter range, was obtained working with 10 kHz repetition rate, 400 fs pulse duration, 75.8 µJ per pulse (Fig. 2C and D). This behavior could be attributed to the generation of cavitation bubbles which could disrupt the NP production mechanism by scattering and absorbing part of the energy deposited by the laser, leading to a less homogenous and less efficient ablation process.^47,48^

Considering these observations, we steered away from the higher repetition rates to obtain more monodispersed NPs. These optimized parameters allowed us producing a NP population suitable for use in electrospun wound dressings. Electron diffraction analysis (Fig. 2E) allowed us to confirm the polycrystalline nature of the SiNPs, as the diffraction rings corresponding to the (111), (220), (222), (311), (331) and (400) planes of the cubic structure of metallic Si were found in our samples, corresponding to the standard pattern provided by the ESRF. The zeta potential of the SiNPs was found to be negative, with a value of −43 ± 2 mV illustrating partial oxidation.

In Fig. 2F, XPS analyses of the freeze-dried SiNPs unveiled the presence of peaks at 98.3 eV (Si2p_3/2_), 98.9 eV (Si2p_1/2_) for Si core atoms (Si^0^) and 103.4 eV (Si2p) for SiO_2_ (Si^4+^) on the surface of the NPs. As the NPs were synthesized in aqueous medium, some amount of surface oxidation is indeed to be expected. However, taking into account the typical thickness probed by XPS with an Al source, (less than 10 nm) the detection of metallic Si on the Si2p spectrum – thus in the topmost layers of the NPs – confirms that the oxide layer is not representative of the whole NP, but rather describes a core–shell structure containing a large metallic core and covered by a thin silicon oxide layer, as we have previously described for NPs produced *via* laser ablation. Corroborated with the electron diffraction pattern described above, we can thus confirm that the NPs produced are mostly consisting of pure, metallic silicon with a thin oxide layer of only few nm.

In view of modulating the amount of SiNPs to be combined with the NFs in the subsequent step of the process, different concentrations of NPs in suspension were prepared. This was carried out by means of centrifugation and controlled supernatant extraction, as described in the experimental section. Three suspensions were thus prepared, referred to as C1, C4 and C16, with the respective NPs concentrations (determined by ICP-MS) of 14; 56 and 224 µg mL^−1^.

### Production and characterization of hybrid NPs/NFs combinations

Taking the pure PCL NF scaffolds as reference, the functionalization of the NFs with the generated SiNPs was explored in a systematic way. To this end, another polymer medium was prepared, replacing the water by the SiNPs suspensions obtained *via* laser ablation. However, these formulations did not allow us to obtain bead-free fibers. Furthermore, the HR-SEM analysis in backscattered electron (BED-C) imaging mode showed that the NPs were not included inside the electrospun NFs. This phenomenon was attributed to the known hydrophobicity of the PCL, which could have prevented the incorporation of the water suspended SiNPs.

Based on these observations, protocol improvement was sought through the addition of a linking moiety for improving the interface between the hydrophobic polymer scaffold and the hydrophilic NPs. Considering the chemical structure of the polymer, and the surface chemistry of the laser synthesized NPs, it was determined that an amino silane would be well suited for this role, as the silane moiety would potentially be able to bond to the SiNPs, and the amine endgroup could possibly play a role in the dispersion of the NPs at the particle/fibers interface.^49,50^ As mentioned in the introduction, we chose APTES to promote the link between the SiNPs and the PCL NFs due to their complementary chemistry. This functionalization step was done in a “one pot” manner and did not complexify the synthesis process.

Building on this strategy, the optimal volume of APTES was determined aiming to maintain ideal electrospinning ability of the polymer melt. Thus, APTES in a volume fraction of 1.7% was added in all subsequent formulations tested below. This was found to be sufficient to produce bead-free fibers in all samples, even in the presence of concentrated suspensions of NPs. We named these formulations P-A-C*x*, where P-A stands for PCL-APTES and *x* is the relative concentration of NPs. Additionally, rheology studies allowed us to observe a sharp decrease in viscosity following the addition of APTES, going from 1934 cP when using pure PCL down to 1130 cP when APTES was added. This relative decrease in viscosity when APTES was employed compared to pure PCL could possibly be attributed to a tensioactive action of this amino silane, favoring the dispersion of the fibers, and limiting inter-fiber interactions. On the other hand, introducing an increasing concentration of SiNPs led to an increase of the suspension viscosity, with 1747, 1836 and 1886 cP for P-A-C1, P-A-C4 and P-A-C16, respectively (Fig. S4). This viscosity rise is consistent with literature observations, as increasing concentrations of NPs in suspension likely leads to increased interactions between particles, hindering fluid movements.^51,52^

**Fig. 3:**
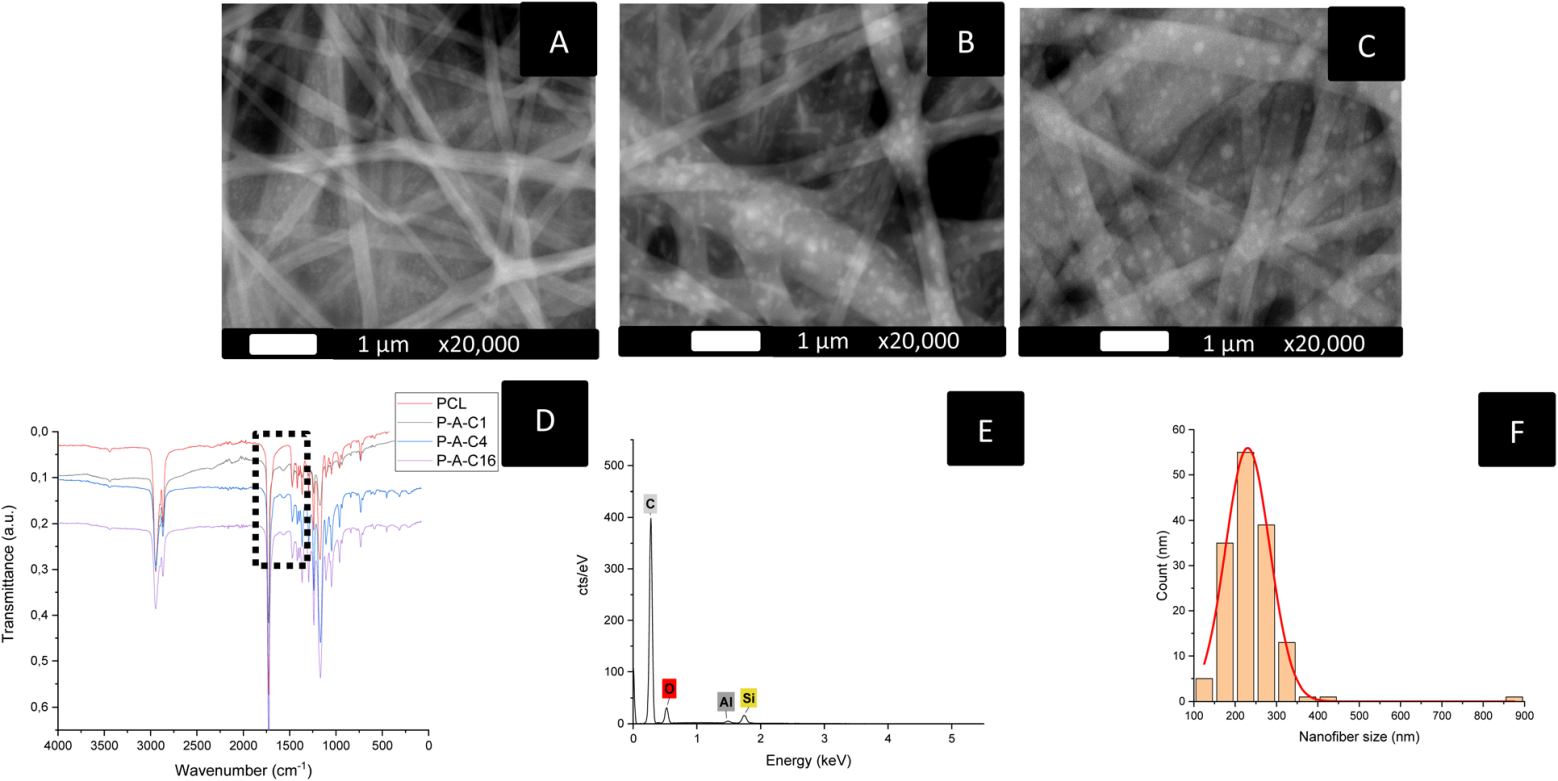
Characterization of the PCL nanofibers functionalized with APTES and laser-synthesized SiNPs. (A): P-A-C1 HR-SEM micrograph in BED-C mode; (B): P-A-C4 HR-SEM micrograph in BED-C mode; (C): P-A-C16 HR-SEM micrograph in BED-C mode (D) comparison of FTIR spectra. (E) EDS spectrum of a P-A-C4 sample. (F) Nanofibers size distribution.

HR-SEM analysis allowed us to observe in detail the obtained hybrid NPs/NFs membranes. Using BED-C imaging, we were able to observe large amounts of SiNPs included in the fibers and well distributed throughout their structure (Fig. 3A–C). NFs obtained when APTES was added to the polymer medium presented a much more homogenous morphology, noticeably without any bead nor other defects. FTIR spectra showed the addition of a characteristic N–H vibration at 1568 cm^−1^, confirming the successful integration of APTES in the nanofiber structure (Fig. 3D). Si was also detected through EDS, illustrating the integration of the NPs in the samples (Fig. 3E). Further exploration of electrospinning parameters using these new formulations led us to reduce the diameter of the nanofibers even further, to an average as low as 245 ± 44 nm when the applied tension was increased to 19 kV (Fig. 3F). These new parameters were used to produce three different formulations of functionalized PCL-APTES-Si-NPs nanofiber wound dressings with increasing concentrations of Si NPs: C1, C4 and C16. These dressings were then subjected to further physico-chemical evaluation, such as porosity, water uptake, stability, and mechanical properties.

### Digital porosity evaluation of the NF samples

**Fig. 4:**
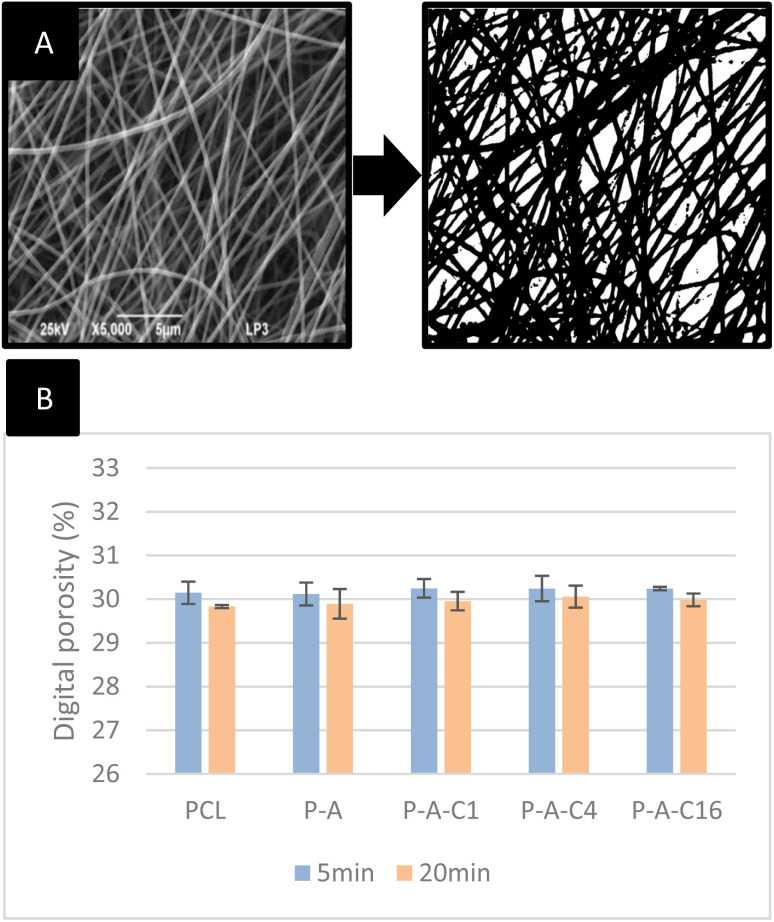
Digital evaluation of the porosity of electrospun nanofibers. (A) Example of 70% thresholding on a P-A-C4 sample micrograph. (B) Digital porosity of the membranes electrospun over 5 or 20 minutes. Asterisks on bars represent a statistically significant variation in porosity when compared to the 5 minutes electrospinning duration (one-factor ANOVA followed by Tukey HSD, *p* < 0.05).

Breathability is a major concern in the development of new wound dressings, as gaseous exchange and moisture management are critical to avoid maceration and thus higher risks of infection. The porosity of our materials was evaluated digitally, through image analysis as described in the experimental section (Fig. 4A). Here, membranes electrospun for 5 to 20 minutes were evaluated and compared. We report no significant variation in porosity over the different formulations of identical electrospinning duration (Fig. 4B). However, we obtain statistical differences between the two sets, with an average porosity of 30.20 ± 0.23% for mats electrospun for 5 min and 29.94 ± 0.20% for mats electrospun during 20 min (*F* = 10.3, *p* = 0.003).

Although the magnitude of this difference remains limited, it is consistent with the expected variation in porosity, as a longer process duration increases the number of nanofiber layers and thus material deposition. Interestingly, these results also show that the chemical composition of the nanofibers – when electrospinning parameters are fixed – is not a major actor in the final porosity of the material. This highly porous structure could present advantageous properties in terms of breathability and moisture management.

### Measurements of structural stability and water uptake of the NFs

**Fig. 5:**
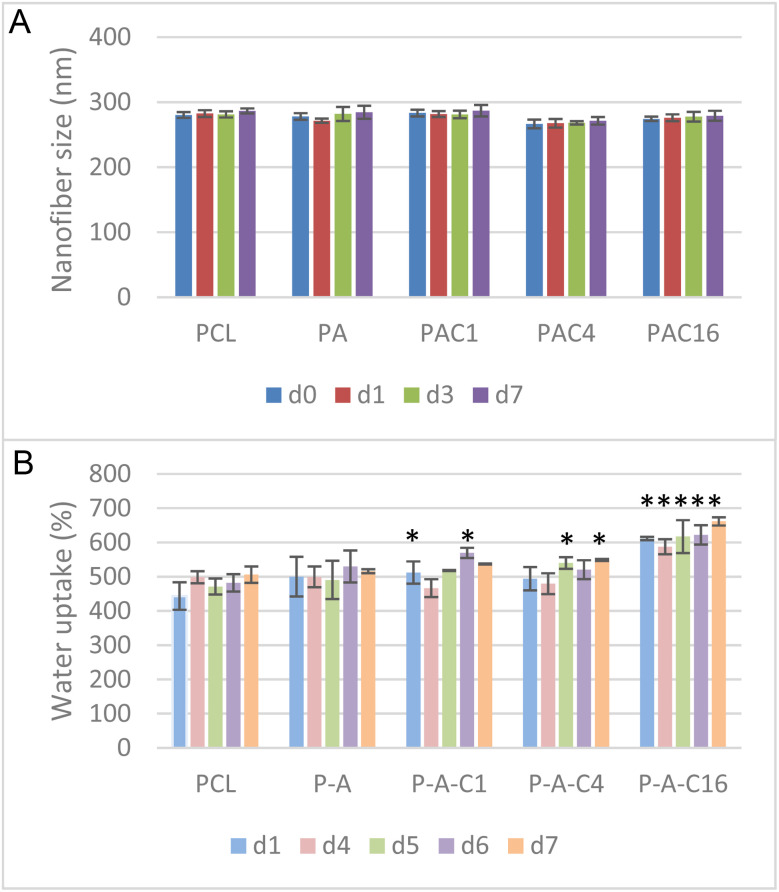
(A) Nanofiber size variation after immersion in PBS for 1–7 days. (B) Water uptake of the nanofibrous membranes during a period of 1–7 days. Asterisks represent a statistical difference between a sample's timepoint and its equivalent for the PCL sample (one-factor ANOVA followed by Tukey HSD, *p* < 0.05).

As stated previously, the developed dressings were further evaluated in PBS to simulate contact with physiological fluids (Fig. 5A) and deionized water to evaluate the water uptake of the NFs (Fig. 5B). The goal of these tests was to assess the NFs membranes usability as a wound-dressing.

In this sense, the dressings were immersed in PBS to evaluate their stability in these conditions. After immersion times of 24 h, 72 h and 7 days, the nanofiber's width, overall structure, and mass were evaluated. These specific timepoints were selected as it is recommended to replace a dressing every 2–3 days, up to 7 days for chirurgical wounds.^53^ The resulting variations in size of the NF membranes are reported in Fig. 5A. We have observed no variations in mass over the course of the experiment. No significant variation in size of the NF dressings was observed, despite a slight increase in diameter (average increase of 5.12 nm) over the course of the 7 days experiment, which remained statistically insignificant.

This slight variation in size could be due to some extent of water absorption in the NF membranes, dilating the NFs.

These results confirm that the PCL membranes can maintain their morphology when immersed in liquid for 7 days. Considering the expected use of these NFs as external wound dressings, and not implantable devices, total immersion is most unlikely. Thus, this experiment confirms the suitability of our NFs for use in bandages or small-scale external dressings.

As a predictive tool for the exudate absorption capabilities of the NFs, we determined the water uptake of NFs when immersed for up to 7 days. The results reported in Fig. 5B show a clear effect of the nanostructure of the material, as even pure PCL NFs – constituted of a hydrophobic material – can absorb between 443 and 506% of their own weight in water over the course of 7 days. The addition of APTES to the formulation slightly increased the water uptake of the membrane, going from 500 to 516%. A similar trend was observed with the increasing concentrations of SiNPs, with water uptakes increasing from 512% to 537% for P-A-C1, 494% to 549% for P-A-C4 and 611% to 661% for P-A-C16 over the duration of the experiment. This evolution can be explained by the synergic effects of multiple phenomena. Firstly, the high surface area and high porosity of the NFs scaffolds help with water sorption, as these nanostructures induce capillary forces that retain water even in a typically hydrophobic material. Consequently, smoother and finer nanofibers should increase the water uptake and hydrophilicity of the dressings, following the Lucas–Washburn equation.^54,55^ Secondly, the grafting of hydrophilic and polar moieties onto nanofibers is a proven method for wettability increase of hydrophobic materials.^56^ By introducing –OH, –COOH or –NH_2_ groups on the surface of the electrospun mats, by the means of plasma treatment,^57^ hydrolysis,^58^ aminolysis^58^ or covalent bonding,^59^ the surface wettability can be modulated. In our case, the addition of APTES introduces –NH_2_ in the PCL matrix, favoring interactions with water and thus increasing the water uptake of the membranes. Thirdly, the introduction of SiNPs in the polymer matrix also seems to significantly increase the water uptake in a dose-dependent manner. The NPs are dispersed in water and present an oxidated layer and surface charges, increasing the favorable interactions with water. This wettability-improving effect of NF functionalization by NP has been reported multiple times not only over various polymers,^60,61^ but also with PCL nanofibers.^62,63^ All these factors are important for the improved properties and water absorption capabilities of the nanofibrous scaffolds.

### Mechanical evaluation of the NF formulations

**Fig. 6:**
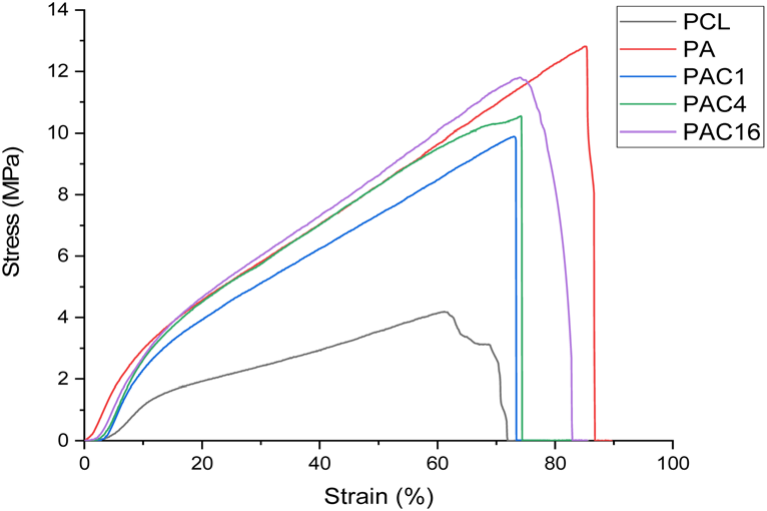
Stress/strain curves of the various NF samples.

Finally, the mechanical resistance of the NF mats was evaluated to assess their suitability for use as a dressing. Thus, the samples' apparent Young modulus, yield modulus and tensile stress at break were evaluated. As shown in Fig. 6, the stress/strain curves were recorded using a tensile testing equipment, and the results are reported in Table 2. We can observe two quasi-linear regions in the stress–strain curves produced. This phenomenon is recurrent in NFs mechanical evaluation, as the mats are constituted of individual fibers and not bulk material.^64,65^ Thus, under low strain, the NFs can rearrange and orient themselves to some extent along the strain orientation.^65,66^ These phenomena explain the qualification of our calculated moduli as apparent, as the cross-sectional area does not take into account the porosity of our nanofibrous material. We describe the modulus calculated in the low strain region, where the usual Young's modulus would be determined, as the apparent Young's modulus. The second linear region, from the first inflexion point to the yield point, can be described as the yield modulus, and was determined from 20 to 60% strain.

**Table 2:** Mechanical properties of the electrospun NFs formulations

	Apparent Young's modulus (MPa)	Yield modulus (MPa)	Maximum load (N)	Stress at break (MPa)	Strain at break (%)
PCL	18 ± 3	5.1 ± 0.5	1.4 ± 0.1	4.3 ± 0.2	66 ± 3
P-A	39 ± 3	12.0 ± 1.1	4.1 ± 0.3	11.9 ± 0.8	83 ± 8
P-A-C1	39 ± 3	11.2 ± 0.7	3.4 ± 0.4	9.9 ± 1.1	75 ± 18
P-A-C4	37 ± 4	12.9 ± 0.8	3.7 ± 0.3	10.7 ± 0.9	75 ± 3
P-A-C16	38 ± 6	13.2 ± 0.8	4.0 ± 0.1	11.7 ± 0.3	76 ± 3

The presence of APTES in the NF mats increases both moduli roughly twofold when compared to pure PCL NFs. Whereas the apparent Young's modulus remains stable when increasing the concentration of SiNPs, the yield modulus seems to increase slightly following the increasing concentration of SiNPs in the NFs. Additionally, stress at break is also increased on average by a factor of 2.6 when APTES is present in the NF mats. This behavior is typical of a reinforcement of the composite material, presenting increased rigidity. Interestingly, we observed a slight decrease in maximum load/stress and strain when comparing the PA sample to those incorporating the SiNPs. Increasing the SiNPs concentration seems to counteract this phenomenon, with values obtained for the PAC16 sample reaching those of the PA sample.

This effect has also been previously described, with low concentrations of NP in NF scaffolds, which can produce localized clusters acting as defects in the polymer structure, thus increasing the stress.^67,68^ Increasing the concentration can lead to the formation of a network of NPs, better able to efficiently distribute stress throughout the fibers.^67,69,70^ Overall, the mechanical properties of the electrospun PCL mats are enhanced by the APTES-SiNPs functionalization.

### Cytocompatibility evaluation of the PCL-APTES-SiNPs formulations using C2C12 cells

Multiple approaches were studied for the biological evaluation of our dressings. In a first stage, we aimed at validating the cytocompatibility of the device *in vitro*. These tests were carried out on murine myoblast C2C12 to assess the possible toxicity of the NFs-NPs hybrid biomaterial in the case of open wounds with direct contact with different types of cells including muscular lineages. Such cells are regularly encountered in the literature, including in the evaluation of wound healing devices.^71–73^ Our previous work on the *in vitro* evaluation of NPs in suspension has also been run with this cell line, granting valuable experience in the experimental setup.^15^

Standard MTT procedure was tested on our nanofibrous membranes. However, unreliable results were obtained due to the absorption of the dissolved formazan on the samples, retaining a characteristic purple color indicative of the impregnation of the fibrous scaffolds by this MTT reactant (Fig. S6). This issue has been described in the literature for different types of fibrous materials including PCL and other polymers, and for different biological assays such as MTT, Alamar blue or others, illustrating the difficulty with the biological evaluation of nanofiber-based materials.^74,75^

**Fig. 7:**
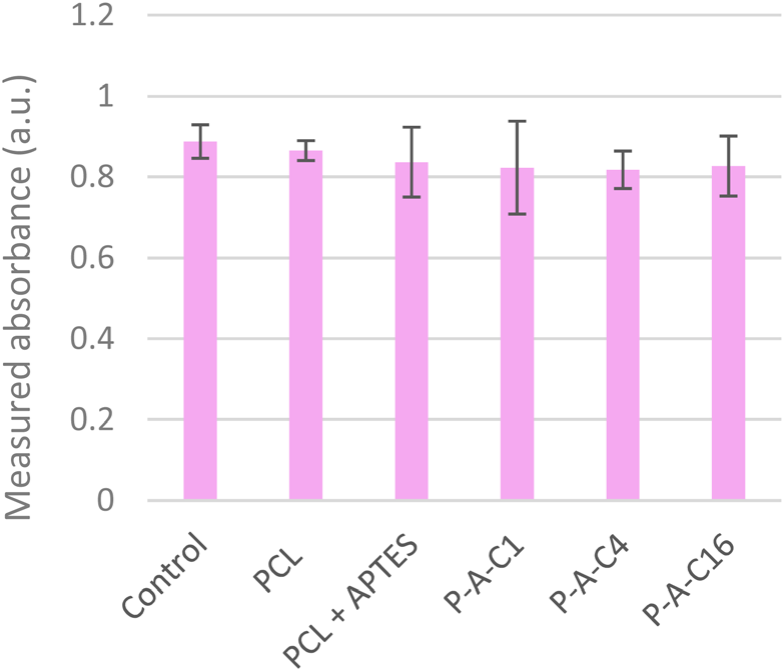
Results of indirect MTT assay (ISO 10993-5) on all nanofibrous biomaterials formulations.

Therefore, as an alternative method, we followed the ISO-10993-5 procedure (Fig. 7), which is a standard in the medical device industry.^34^

This approach relies on an indirect cytotoxicity assay, where DMEM culture medium is incubated for 24 hours in the presence of the tested material before being transferred to pre-seeded cells.

By conducting the MTT assay in this manner, we can avoid interference from the absorption issue on electrospun nanofibers. If toxic compounds, such as released NPs or ions, or else nanofiber fragments were present in the medium, their effects on the overall cytocompatibility could then be quantified.

Each nanofiber film was cut into 2 × 2 cm squares and carefully withdrawn from the aluminum foil on which they were deposited during the electrospinning process. Each sample was weighed to ensure no significant variation in material quantity. They were then placed into the culture wells, secured with custom 3D-printed biocompatible PLA inserts to maintain the samples planarity and control their flotation in the wells. Culture medium was placed in the wells and put to incubation for 24 h. The extracted medium was then applied to the C2C12 cells, and viability was assessed *via* the MTT assay after 48 hours of exposure, in accordance with the ISO 10993-5 guidelines. Interestingly, no significant difference was observed between the samples and the control (consisting in cells cultivated in classical DMEM medium), confirming the absence of any toxicity related to the nanofibers, NPs, or their derivatives.

### Biological evaluation of the PCL-APTES-SiNPs formulations using HaCat cells

Additional *in vitro* evaluation was then carried out human keratinocytes HaCaT, in order to extend our biocompatibility assessments to a human lineage and also to undergo tests on a key cell type of the skin, taking into account the theoretical external usage of the dressings.

**Fig. 8:**
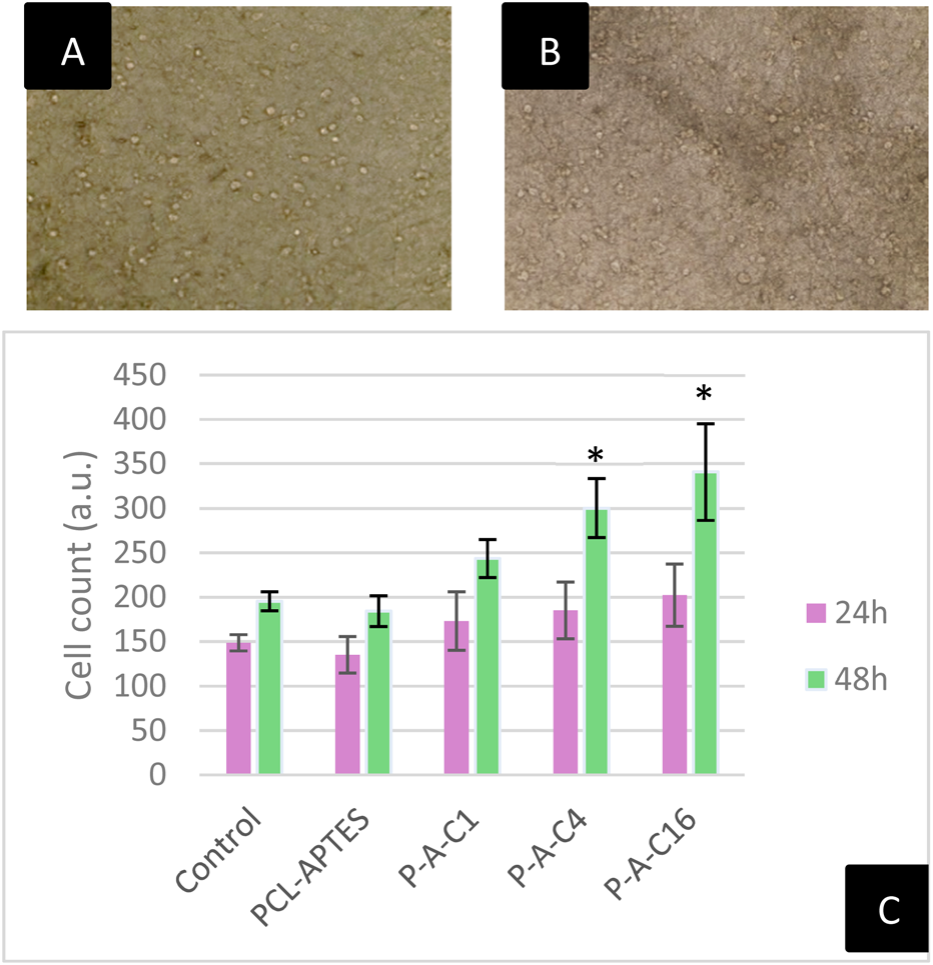
Inverted microscope pictures of HaCaT cells seeded onto the nanofibrous membranes, 10× magnification: (A) PCL-APTES at 48 h and (B) P-A-C16 at 48 h. (C) Cell counts of HaCaT cells on nanofibrous membranes through direct observation at 24 and 48 h. Asterisks above the error bars represent statistical difference when compared to control (one-factor ANOVA followed by Tukey HSD, *p* < 0.05).

Due to the previously described technical challenges in the biological evaluation of our fibrous formulations, reagent-based or staining methods, such as CellTiter-Glo or Hoechst staining, were not suitable for evaluating our samples. Instead, we opted for cell counting, through direct observation of the cells on the dressings (Fig. 8).

By seeding 50 000 keratinocytes onto the nanofibrous samples, cell outlines were clearly visible under an inverted microscope (Fig. 8A and B), allowing us to estimate cell numbers and assess keratinocyte proliferation on the dressings. This could be done for the APTES-modified PCL fibers with or without associated SiNPs. The results then pointed out the normal cell morphology on the scaffolds, inferring the absence of noticeable detrimental effect on the cells. Note that in the case of pure PCL (without APTES modification), the structure of the nanofibrous dressings was more opaque and did not provide sufficient transparency for reliable cell counting. However, since our experiments above showed no difference between pure PCL and PCL-APTES in terms of cell survival on C2C12 cells, no specific issue could reasonably be stated.

As Fig. 8C shows, 24 h after seeding, the cell count on the PCL-APTES sample (thus without NPs) was slightly lower than in the control, though this decrease was not statistically significant. The nanofibrous scaffolds containing Si-NPs exhibited in contrast a slightly higher cell count compared to the control, but again not in a statistically significant manner.

This first timepoint confirmed that cells could adhere to and survive on the nanofibrous membranes upon initial contact, with no negative impact from APTES or SiNPs in the different formulations.

At 48 h, a relative increase in cell numbers was observed across all formulations. Between 24 and 48 h, the control wells showed an average increase of 32%, while PCL-APTES increased by 36%, P-A-C1 by 41%, P-A-C4 by 62%, and P-A-C16 by 69%. The cell counts on PCL-APTES remained slightly lower than in the control, as expected, but within an acceptable range for biocompatibility. Interestingly, all Si-NPs-containing samples exhibited significantly higher cell numbers than the control, with the effect increasing proportionally to the NPs concentration. Notably, P-A-C16 produced the highest proliferation, with nearly twice as many cells counted on its surface compared to the control. These results confirm the previous observations, mainly that APTES is safe to use in a limited quantity in the wound dressing, and our results show that increasing concentrations of laser-synthesized Si-NPs drastically improves cell proliferation. The understanding of this phenomenon is of importance, as contemporary literature mainly focuses on silicon oxides as a source of biological activity. In the context of laser synthesized NPs, although the extreme outermost layer of the material is oxidized, most of the NP is composed of pure elemental silicon. Laser synthesized SiNPs have been unveiled as a promising material for theranostics but have not been fully explored as a proper growth-inducing alternative for tissue engineering. In some of our lab's previous work, these NP have been described as promoting murine cell growth and differentiation, shedding light on their potential for wound healing applications.^15^ The mechanism of action is still elusive, and requires further exploration to fully grasp the potential of laser synthesized SiNPs. In this cited study, murine C2C12 gene expression was measured and markers of proliferation Ki-67, mobility C-met and muscle differentiation were found to be overexpressed, illustrating the effect on cellular machinery.

**Fig. 9:**
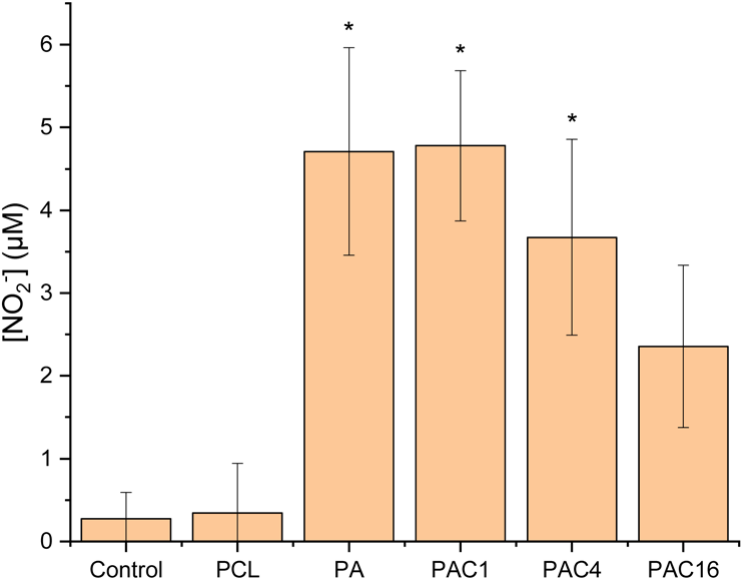
Griess inflammation assay results on human keratinocytes (HaCaT) in contact to different nanofibrous membranes prepared with increasing Si-NPs contents. Asterisks above the error bars represent statistical difference when compared to control (one-factor ANOVA followed by Tukey HSD, *p* < 0.05).

In a second stage, we aimed at evaluating the potential inflammatory response of our developed nanofibrous wound dressings, including our best formulation in terms of proliferative potential, namely the P-A-C16 sample. To this end, we used the established Griess assay.^71,76,77^ In this test, the nitric oxide (NO) released from cells under inflammatory stress is detected and quantified photometrically in the nitrite form upon reaction with the Griess reagent. Although HaCaT cells are keratinocytes and therefore cannot fully replicate the entire inflammatory response of all skin cell types and immunity cells, they can provide a relevant model to assess initial inflammatory response of epidermal cells, *i.e.* the first cells in prolonged contact with the dressing. The HaCaT cells were cultured onto the nanofibrous membranes, and after 48 h of incubation the culture medium was sampled and tested for the presence of NO using the Griess reagent and compared to a previously established calibration curve. This assay (Fig. 9) uncovered the presence of minute amounts of NO released when cells were put in contact with the nanofibrous samples containing APTES (with or without Si-NPs), reaching a maximum of 4.6 µM in the PCL-APTES (PA) sample. This value is found well within homeostatic values (<8–10 µM),^78–80^ thus illustrating the non-proinflammatory potential of all of our developed wound dressings. Furthermore, previously obtained cytocompatibility assays showed no statistically significant difference between pure PCL and P-A samples, supporting the idea of non-detrimental NO levels.

We may also note that a decrease of the detected nitrite concentration was observed upon increasing the SiNPs concentration in the samples, down to a concentration as low as 2.3 µM, which seems to correspond to an increased proliferation and activation of cell survival mechanisms, and could be linked to the bioactivity of the SiNPs themselves.^80–84^

## Conclusion

In this contribution, we developed various formulations of innovative bioactive wound dressings composed of a network of imbricated polymeric nanofibers (ε-PCL), possibly functionalized with APTES, and associated with silicon (Si) NPs. In our approach, such SiNPs have been prepared using a “green” laser-based/solvent-free (PLAL) technology. In the hybrid NFs-NPs wound dressings obtained, the SiNPs were found to be well distributed throughout the fibers structure without forming clotting or clustered heterogeneous domains, allowing us to increase the SiNPs concentration on-demand. We thus ran a systematic investigation to determine the role of different compositional and process parameters, with the aim to identify optimized production conditions. The physico-chemical characterization of the PCL fibers with or without NPs or APTES was undergone by FTIR, indicating that the polymer was not altered during the production process. The addition of APTES onto the PCL nanofibers clearly enhanced the homogeneity, mechanical properties and water absorption of the fibers, and yielded lower fiber diameters around 245 nm. The different formulations were not found to be degraded or morphologically affected upon immersion in PBS for up to 7 days, illustrating their robustness and potential usability as a wound dressing. Their biocompatibility as well as the specific biological role of the particles themselves were verified *in vitro* using two cell types, namely C2C12 myoblasts and HaCaT keratinocytes. A proliferative effect was also underlined for the NPs and proportionally with their concentration in the device. Griess tests finally highlighted reasonable inflammatory response for all the biomaterials tested, and mitigation of the inflammation by the increasing concentration of NPs was even noticed.

Taking into account the biocompatibility of the dressings, produced by coupling the “green” electrospinning and laser ablation methods, we anticipate that such hybrid bioactive devices could allow progressing in the management of complex wounds.

## Author contributions

Conceptualization: AAK, RS. Data curation: AAK, CD, MGB, RS, CN, DD, JE, MA. Formal analysis: RS. Funding acquisition: AAK. Investigation: AAK, CD, MGB, RS, CN, DD, JE, MA. Methodology: AAK, RS, MGB. Supervision: AAK, CD, MGB. Writing – original draft and editing: AAK, CD, MGB, RS, CN, DD, JE, MA.

## Conflicts of interest

There are no conflicts to declare.

## Supplementary Material

RA-016-D5RA09481J-s001

## Data Availability

The data supporting this article have been included as part of the manuscript and the supplementary information (SI). Supplementary information is available. See DOI: https://doi.org/10.1039/d5ra09481j.

## References

[cit1] Sood A., Granick M. S., Tomaselli N. L. (2014). Adv. Wound Care.

[cit2] Weller C. D., Team V., Sussman G. (2020). Front. Pharmacol..

[cit3] Gao C., Zhang L., Wang J., Jin M., Tang Q., Chen Z., Cheng Y., Yang R., Zhao G. (2021). J. Mater. Chem. B.

[cit4] Hromadka M., Collins J. B., Reed C., Han L., Kolappa K. K., Cairns B. A., Andrady T., Van Aalst J. A. (2008). J. Burn Care Res..

[cit5] Lu X., Li X., Yu J., Ding B. (2022). Acta Biomater..

[cit6] Kahdim Q. S., Abdelmoula N., Al-Karagoly H., Albukhaty S., Al-Saaidi J. (2023). BioTech.

[cit7] Su Y., Wang H., Mishra B., Lakshmaiah Narayana J., Jiang J., Reilly D. A., Hollins R. R., Carlson M. A., Wang G., Xie J. (2019). Mol. Pharm..

[cit8] Wróblewska-Krepsztul J., Rydzkowski T., Michalska-Pożoga I., Thakur V. K. (2019). Nanomaterials.

[cit9] Raina N., Pahwa R., Khosla J. K., Gupta P. N., Gupta M. (2022). Polym. Bull..

[cit10] Joseph B., Augustine R., Kalarikkal N., Thomas S., Seantier B., Grohens Y. (2019). Mater. Today Commun..

[cit11] Sun H., Mei L., Song C., Cui X., Wang P. (2006). Biomaterials.

[cit12] Augustine R., Dominic E. A., Reju I., Kaimal B., Kalarikkal N., Thomas S. (2015). J. Biomed. Mater. Res., Part B.

[cit13] Mokhena T. C., Chabalala M. B., Mapukata S., Mtibe A., Hlekelele L., Cele Z., Mochane M. J., Ntsendwana B., Nhlapo T. A., Mokoena T. P., Bambo M. F., Matabola K. P., Ray S. S., Sadiku E. R., Shingange K. (2024). Macromol. Mater. Eng..

[cit14] Rane A. V. , Kanny K., Abitha V. K. and Thomas S., in Synthesis of Inorganic Nanomaterials, ed. S. Mohan Bhagyaraj, O. S. Oluwafemi, N. Kalarikkal and S. Thomas, Woodhead Publishing, 2018, pp. 121–139

[cit15] Murru C., Duvert L., Magdinier F., Casanova A., Alloncle A.-P., Testa S., Al-Kattan A. (2024). Nanoscale Adv..

[cit16] Correard F., Maximova K., Estève M.-A., Villard C., Roy M., Al-Kattan A., Sentis M., Gingras M., Kabashin A. V., Braguer D. (2014). Int. J. Nanomed..

[cit17] Al-Kattan A., Tselikov G., Metwally K., Popov A. A., Mensah S., Kabashin A. V. (2021). Nanomaterials.

[cit18] Al-Kattan A., Ryabchikov Y. V., Baati T., Chirvony V., Sánchez-Royo J. F., Sentis M., Braguer D., Timoshenko V. Y., Estève M.-A., Kabashin A. V. (2016). J. Mater. Chem. B.

[cit19] Al-Kattan A., Ali L. M. A., Daurat M., Mattana E., Gary-Bobo M. (2020). Nanomaterials.

[cit20] Quignard S., Coradin T., Powell J. J., Jugdaohsingh R. (2017). Colloids Surf., B.

[cit21] Seaborn C. D., Nielsen F. H. (2002). Biol. Trace Elem. Res..

[cit22] Mathew-Steiner S. S., Roy S., Sen C. K. (2021). Bioengineering.

[cit23] Xue M., Jackson C. J. (2015). Adv. Wound Care.

[cit24] Schultz G. S. , Chin G. A., Moldawer L. and Diegelmann R. F., in Mechanisms of Vascular Disease: A Reference Book for Vascular Specialists, ed. R. Fitridge and M. Thompson, University of Adelaide Press, Adelaide (AU), 2011 30484990

[cit25] Saikia J., Mohammadpour R., Yazdimamaghani M., Northrup H., Hlady V., Ghandehari H. (2018). ACS Appl. Bio Mater..

[cit26] Yohai L., Mejía H. G., Procaccini R., Pellice S., Kunjali K. L., Dutta J., Uheida A. (2019). RSC Adv..

[cit27] Keshtkar A. R., Tabatabaeefar A., Vaneghi A. S., Moosavian M. A. (2016). J. Environ. Chem. Eng..

[cit28] Meng H., Li X., Wang C., Meng H., Li S. (2025). J. Environ. Chem. Eng..

[cit29] Kurniawan A., Gunawan F., Nugraha A. T., Ismadji S., Wang M.-J. (2017). Int. J. Pharm..

[cit30] Shahriari-Khalaji M., Hu G., Chen L., Cao Z., Andreeva T., Xiong X., Krastev R., Hong F. F. (2021). ACS Biomater. Sci. Eng..

[cit31] Delyanee M., Solouk A., Akbari S., Daliri Joupari M. (2021). Polym. Adv. Technol..

[cit32] Salim S. A., Loutfy S. A., El-Fakharany E. M., Taha T. H., Hussien Y., Kamoun E. A. (2021). J. Drug Delivery Sci. Technol..

[cit33] Cuahuizo-Huitzil G., Olivares-Xometl O., Arellanes-Lozada P., Laguna Cortés J. O., Arriola Morales J., Santacruz-Vázquez C., Santacruz-Vázquez V. (2024). Polymers.

[cit34] ISO 10993-5 , https://www.iso.org/fr/standard/36406.html, accessed 31 January 2025

[cit35] Al-Kattan A., Nirwan V. P., Munnier E., Chourpa I., Fahmi A., Kabashin A. V. (2017). RSC Adv..

[cit36] Nirwan V. P., Al-Kattan A., Fahmi A., Kabashin A. V. (2019). Nanomaterials.

[cit37] Zuo W., Zhu M., Yang W., Yu H., Chen Y., Zhang Y. (2005). Polym. Eng. Sci..

[cit38] Okutan N., Terzi P., Altay F. (2014). Food Hydrocolloids.

[cit39] Demir M. M., Yilgor I., Yilgor E., Erman B. (2002). Polymer.

[cit40] Bhardwaj N., Kundu S. C. (2010). Biotechnol. Adv..

[cit41] Azizi M., Azimzadeh M., Afzali M., Alafzadeh M., Mirhosseini S. (2018). Journal of Advanced Materials and Processing.

[cit42] Bulbul Y. E., Dilsiz N. (2024). Fibers Polym..

[cit43] Al-Kattan A., Ryabchikov Y. V., Baati T., Chirvony V., Sánchez-Royo J. F., Sentis M., Braguer D., Timoshenko V. Y., Estève M.-A., Kabashin A. V. (2016). J. Mater. Chem. B.

[cit44] Kong B., Seog J. H., Graham L. M., Lee S. B. (2011). Nanomedicine.

[cit45] Dong X., Wu Z., Li X., Xiao L., Yang M., Li Y., Duan J., Sun Z. (2020). Int. J. Nanomed..

[cit46] Sahu D., Kannan G. M., Tailang M., Vijayaraghavan R. (2016). J. Nanosci..

[cit47] Sajti C. L., Sattari R., Chichkov B. N., Barcikowski S. (2010). J. Phys. Chem. C.

[cit48] Wagener P., Schwenke A., Chichkov B. N., Barcikowski S. (2010). J. Phys. Chem. C.

[cit49] Bunnett J. F., Davis G. T. (1960). J. Am. Chem. Soc..

[cit50] Eslami S., Farhangdoost B., Shahverdi H., Mohammadi M. (2021). Greenhouse Gases:Sci. Technol..

[cit51] Chen H., Witharana S., Jin Y., Kim C., Ding Y. (2009). Particuology.

[cit52] Yalçın G., Öztuna S., Dalkılıç A. S., Wongwises S. (2024). J. Therm. Anal. Calorim..

[cit53] Pansements pour plaies suturées, à la suite d’une intervention chirurgicale – Fiche BUTS , https://www.has-sante.fr/jcms/p_3394796/fr/pansements-pour-plaies-suturees-a-la-suite-d-une-intervention-chirurgicale-fiche-buts, accessed 14 October 2025

[cit54] Tiyek I., Gunduz A., Yalcinkaya F., Chaloupek J. (2019). J. Nanosci. Nanotechnol..

[cit55] Cai J., Jin T., Kou J., Zou S., Xiao J., Meng Q. (2021). Langmuir.

[cit56] Niemczyk-Soczynska B., Gradys A., Sajkiewicz P. (2020). Polymers.

[cit57] Esmaeili Ranjbar A., Asadi F., Mohandesnezhad S., Vatanparast M., Mohandesnezhad S., Mirzaei M. R., Noroozi Karimabad M., Fathabadi A. S., Esmaeili Ranjbar F. (2025). Sci. Rep..

[cit58] Yaseri R., Fadaie M., Mirzaei E., Samadian H., Ebrahiminezhad A. (2023). Sci. Rep..

[cit59] Wu C.-S. (2005). Polymer.

[cit60] Yang Z., Guo H., Yao Z., Mei Y., Tang C. Y. (2019). Environ. Sci. Technol..

[cit61] Pereira F. A. S., Salles G. N., Rodrigues B. V. M., Marciano F. R., Pacheco-Soares C., Lobo A. O. (2016). Mater. Lett..

[cit62] Mosallanezhad P., Nazockdast H., Ahmadi Z., Rostami A. (2022). Front. Bioeng. Biotechnol..

[cit63] Ghosal K., Manakhov A., Zajíčková L., Thomas S. (2017). AAPS PharmSciTech.

[cit64] Mubyana K., Koppes R. A., Lee K. L., Cooper J. A., Corr D. T. (2016). J. Biomed. Mater. Res., Part A.

[cit65] Maccaferri E., Cocchi D., Mazzocchetti L., Benelli T., Brugo T. M., Giorgini L., Zucchelli A. (2021). Macromol. Mater. Eng..

[cit66] Verschatse O., Loccufier E., Swanckaert B., De Clerck K., Daelemans L. (2023). Polymers.

[cit67] Zaccaria M., Gualandi C., Fabiani D., Focarete M. L., Croce F. (2012). J. Nanomater..

[cit68] Owoyemi H. T., Adewuyi B. O., Oladele I. O., Falana S. O., Oyegunna S. A., Ajileye J. O. (2024). Discov. Polym..

[cit69] Li X., Li Z., Shen J., Zheng Z., Liu J. (2021). Phys. Chem. Chem. Phys..

[cit70] Shen J., Lin X., Liu J., Li X. (2020). Phys. Chem. Chem. Phys..

[cit71] Bui H. T., Chung O. H., Dela Cruz J., Park J. S. (2014). Macromol. Res..

[cit72] Najafi S., Barasa L., Frianela J. M. J., Alkhamisy J. H., Yoganathan S., Perron J. C. (2023). FBL.

[cit73] Park S. Y., Lee H. U., Lee Y.-C., Kim G. H., Park E. C., Han S. H., Lee J. G., Choi S., Heo N. S., Kim D. L., Huh Y. S., Lee J. (2014). Mater. Sci. Eng., C.

[cit74] Qi R., Shen M., Cao X., Guo R., Tian X., Yu J., Shi X. (2011). Analyst.

[cit75] Podgórski R., Wojasiński M., Ciach T. (2022). Sci. Rep..

[cit76] Schmölz L., Wallert M., Lorkowski S. (2017). J. Immunol. Methods.

[cit77] Sun J., Zhang X., Broderick M., Fein H. (2003). Sensors.

[cit78] Tejedo J. R., Tapia-Limonchi R., Mora-Castilla S., Cahuana G. M., Hmadcha A., Martin F., Bedoya F. J., Soria B. (2010). Cell Death Dis..

[cit79] Culmsee C., Gerling N., Landshamer S., Rickerts B., Duchstein H.-J., Umezawa K., Klumpp S., Krieglstein J. (2005). Mol. Pharmacol..

[cit80] Frank S., Kämpfer H., Wetzler C., Pfeilschifter J. (2002). Kidney Int..

[cit81] Man M.-Q., Wakefield J. S., Mauro T. M., Elias P. M. (2022). Exp. Dermatol..

[cit82] Luo J., Chen A. F. (2005). Acta Pharmacol. Sin..

[cit83] Malone-Povolny M. J., Maloney S. E., Schoenfisch M. H. (2019). Adv. Healthcare Mater..

[cit84] Wu M., Lu Z., Wu K., Nam C., Zhang L., Guo J. (2021). J. Mater. Chem. B.

