# Supplementary material for: Combination of laser-generated silicon NPs and electrospun-nanofibres for the development of new-generation bioactive wound dressings
Source: RSC Adv. 2026 Jan 2;16(1):604–16. doi: 10.1039/d5ra09481j (PMC12757934; doi:10.1039/d5ra09481j)
Supplement: RA-016-D5RA09481J-s001 [file RA-016-D5RA09481J-s001.pdf]

Supplementary Information (SI) for RSC Advances.
This journal is © The Royal Society of Chemistry 2025

Combination of laser-generated Silicon nanoparticles and electrospun-nanofibers for the development of new-generation bioactive wound dressings

Romain Scarabelli[1,2], Magali Gary-Bobo[3], Christophe Nguyen[3], Denis Durand[3], Jérôme Esvan[2], Maëlenn Aufray[2] , Christophe Drouet[2], Ahmed Al-Kattan[1]*

1- LP3, Aix Marseille Université, CNRS, UMR 7341, Campus de Luminy, Case 917,13288, Marseille, France
2- CIRIMAT, Université de Toulouse/INP/CNRS, 4 allée Emile Monso, 31030 Toulouse, France
3- IBMM, Université de Montpellier, CNRS, ENSCM, 1919 Route de Mende, 34293 Montpellier, France

Corresponding author: ahmed.al-kattan@univ-amu.fr

Supplementary Information

Production and characterization of PCL nanofibers

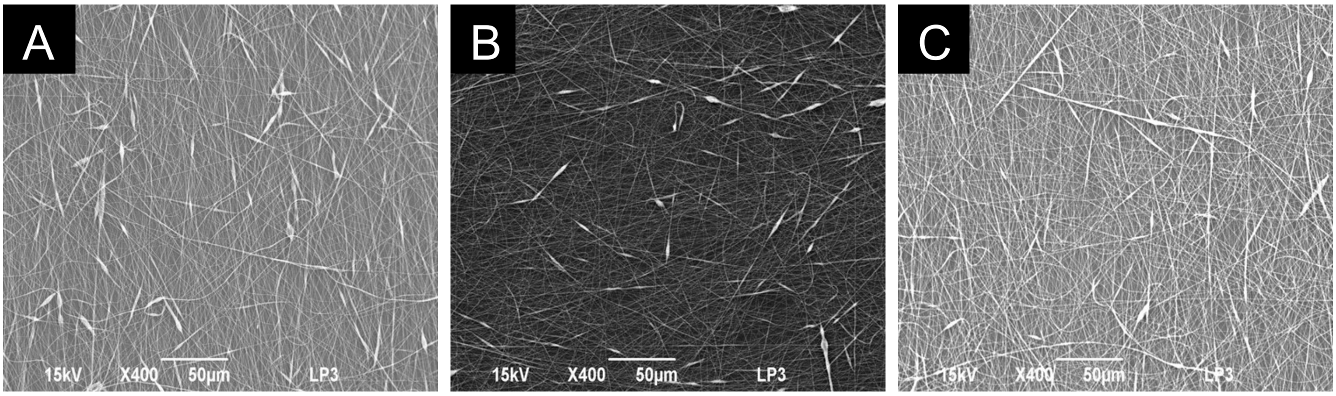

Figure S1: SEM micrographs for various electrospinning parameters during the optimization process. (A): 10 kV 13.75 cm; (B): 13.5 kV 18 cm; (C): 20 kV 14.25 cm

Production and characterization of laser-synthesized Silicon NPs

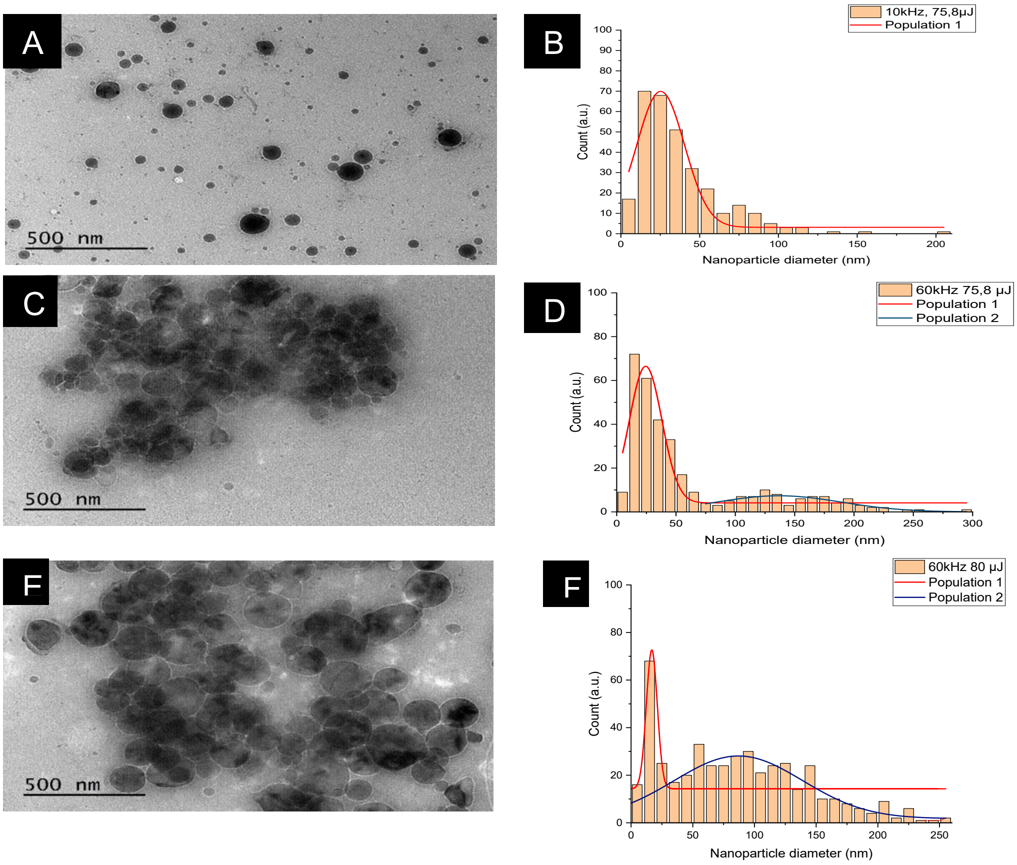

Figure S2: Size distributions and TEM analysis of Si-NPs produced via laser ablation in water. (A): TEM photograph of Si-NPs obtained with 10 kHz, 75.8 µJ; (B): Size distribution of Si-NPs obtained with 10 kHz, 75.8 µJ; (C): TEM photograph of Si-NPs obtained with 60 kHz, 75.8 µJ; (D) Size distribution of Si-NPs obtained with 60 kHz, 75.8 µJ; (E): TEM photograph of Si-NPs obtained with 60 kHz, 80 µJ; (F): Size distribution of Si-NPs obtained with 60 kHz, 80 µJ;

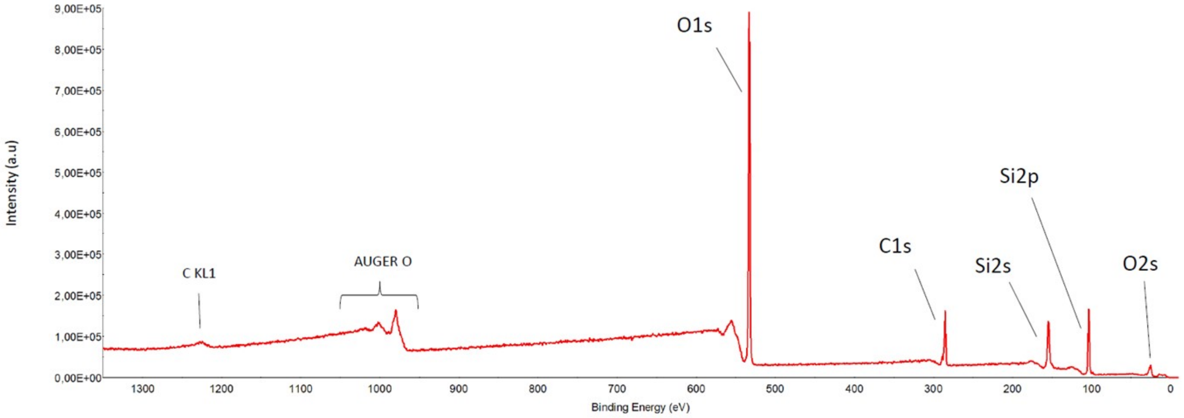

Figure S3: XPS survey of the SiNPs

Production and characterization of hybrid NPs/NFs combinations

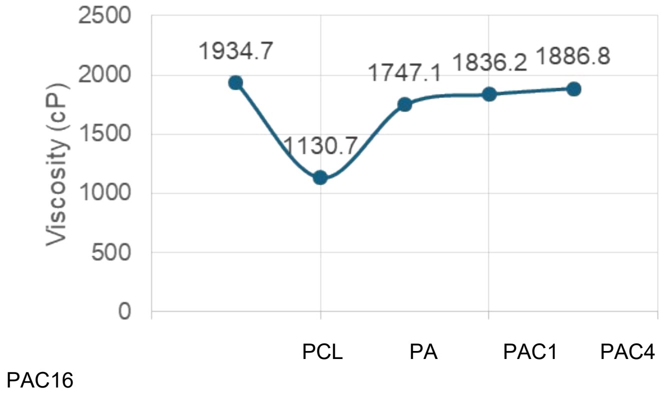

Figure S4: Rheology values of polymer solutions (pre-electrospinning) of all formulations

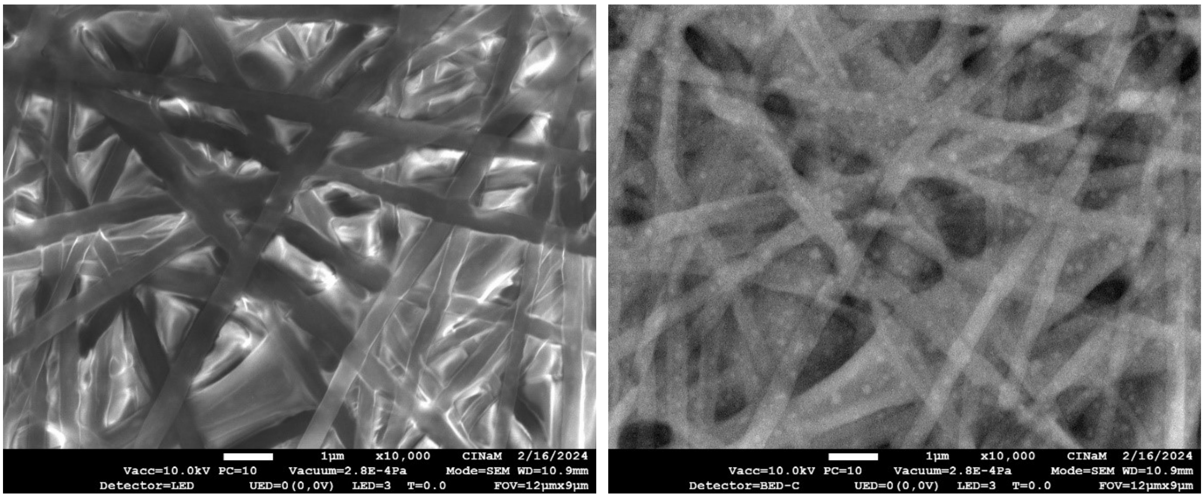

Figure S5: Comparison between LED and BED-C imaging for the detection of SiNPs in a PAC4 sample.

Cytocompatibility evaluation of the PCL-APTES-SiNPs formulations using C2C12 cells

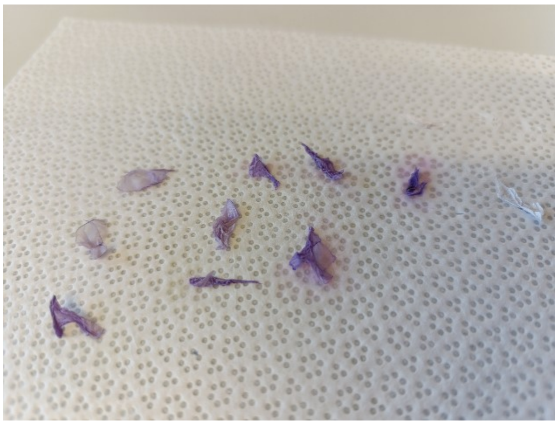

Figure S6: Photograph of NF samples after a direct MTT assay. Membranes were washed and triturated multiple times with isopropanol.
